# *cis*-Diaqua­bis(2,2′,2′′-tripyridylamine)zinc(II) bis­(perchlorate)

**DOI:** 10.1107/S1600536809042688

**Published:** 2009-10-23

**Authors:** Shi Wang, Xuehua Ding, Wenrui He, Wei Huang

**Affiliations:** a Jiangsu Key Lab of Organic Electronics & Information Displays, Institute of Advanced Materials (IAM), Nanjing University of Posts and Telecommunications, Nanjing 210046, People’s Republic of China

## Abstract

In the title compound, [Zn(2,2′,2′′-tpa)_2_(H_2_O)_2_](ClO_4_)_2_ (2,2′,2′′-tpa is 2,2′,2′′-tripyridylamine, C_15_H_12_N_4_), the Zn center lies on a twofold axis and is coordinated octa­hedrally by two water mol­ecules and two bidentate 2,2′,2′′-tpa ligands. The perchlorate anions are linked to the coordinated water mol­ecules in the complex cations *via* O—H⋯O hydrogen bonds.

## Related literature

For general background, see: Liu *et al.* (1997 ▶). For related structures, see: Yang *et al.* (1999 ▶). 
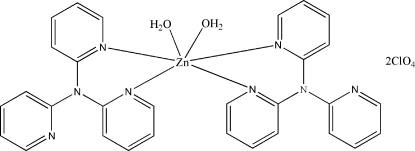


## Experimental

### 

#### Crystal data

[Zn(C_15_H_12_N_4_)_2_(H_2_O)_2_](ClO_4_)_2_
*M* *_r_* = 796.89
Monoclinic, 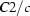

*a* = 18.687 (3) Å
*b* = 19.305 (4) Å
*c* = 10.8910 (19) Å
β = 121.689 (3)°
*V* = 3343.2 (11) Å^3^
*Z* = 4
Mo *K*α radiation
μ = 0.96 mm^−1^
*T* = 200 K
0.35 × 0.22 × 0.20 mm

#### Data collection

Bruker SMART CCD area-detector diffractometer
Absorption correction: multi-scan (*SADABS*; Sheldrick, 1996 ▶) *T*_min_ = 0.775, *T*_max_ = 0.825
7752 measured reflections
2940 independent reflections
1861 reflections with *I* > 2σ(*I*)
*R*_int_ = 0.068

#### Refinement

*R*[*F* ^2^ > 2σ(*F* ^2^)] = 0.057
*wR*(*F* ^2^) = 0.143
*S* = 0.92
2940 reflections
239 parameters
2 restraints
H atoms treated by a mixture of independent and constrained refinement
Δρ_max_ = 0.86 e Å^−3^
Δρ_min_ = −0.41 e Å^−3^

### 

Data collection: *SMART* (Bruker, 2007 ▶); cell refinement: *SAINT* (Bruker, 2007 ▶); data reduction: *SAINT*; program(s) used to solve structure: *SHELXTL* (Sheldrick, 2008 ▶); program(s) used to refine structure: *SHELXTL*; molecular graphics: *DIAMOND* (Brandenburg, 1999 ▶); software used to prepare material for publication: *SHELXTL*.

## Supplementary Material

Crystal structure: contains datablocks I, global. DOI: 10.1107/S1600536809042688/nk2005sup1.cif

Structure factors: contains datablocks I. DOI: 10.1107/S1600536809042688/nk2005Isup2.hkl

Additional supplementary materials:  crystallographic information; 3D view; checkCIF report

## Figures and Tables

**Table 1:** Hydrogen-bond geometry (Å, °)

*D*—H⋯*A*	*D*—H	H⋯*A*	*D*⋯*A*	*D*—H⋯*A*
O1*W*—H2*W*⋯O5^i^	0.83 (2)	2.23 (4)	2.939 (6)	143 (6)
O1*W*—H1*W*⋯O4^ii^	0.85 (4)	2.07 (5)	2.868 (6)	158 (6)

Symmetry codes: (i) 
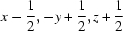
; (ii) 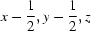.

**Fig. 1.**
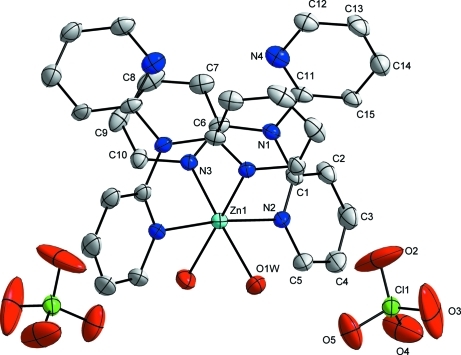
The molecular structure of (I), shown with 30% probability displacement ellipsoids.The unlabeled atoms are derived from the reference atoms by means of the (-x + 1/2, -y + 1/2, -z) symmetry transformation.

**Fig. 2.**
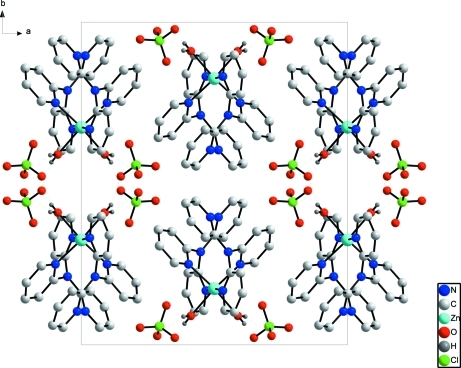
Packing diagram viewed down the c axis, The O—H···O hydrogen bonds are shown as dotted lines.

## supplementary crystallographic information

### Comment

Luminescent organic and coordination compounds have been an active research area for decades because of their various potential applications in materials sciences. It has been demonstrated that 2,2'-dipyridylamine can produce a bright blue luminescence when deprotonated and bound to either an aluminium ion or a boron center (Liu *et al.*, 1997). However, many of the previously reported aluminium or boron compounds based on 2,2'-dipyridylamine are not stable enough for electroluminescent devices. By using the neutral ligand, 2,2',2''-tripyridylamine, we report here the synthesis and crystal structure of the title compound, diaquabis(2,2',2''-tripyridylamine)zinc(II) bis(perchlorate).

The structure consists of monomeric [Zn(2,2',2''-tpa)_2_(H_2_O)_2_]^2+^ cations and associated ClO_4_^-^ anions. The Zn atom lies on a two fold axis. As shown in Fig. 1,the zinc center is six-coordinate with an octahedral geometry. In principle, 2,2',2''-tpa can function not only as a bidentate chelating ligand but also as a tridentate chelating ligand where all three pyridyl groups bind to the same central atom (Yang *et al.*, 1999). In the title compound, each 2,2',2''-tpa ligand functions as a bidentate ligand, chelating to the zinc center. Two water molecules are coodinated to the zinc center as terminal ligands in a *cis* geometry. Crystals of the *trans* geometric isomer of I were not obtained.

The displacement parameters of the perchlorate anion are large. A disorder model has been tried but no improvement in refinement was observed.

Coordinated water molecules in the complex cations are connected to ClO_4_^-^ anions through O—H···O hydrogen bonds (Fig. 2).

### Experimental

The ligand, 2,2',2''-tpa was synthesized according to the procedure described in the literature (Yang *et al.* (1999)).

A solution of 2,2',2''-tpa (62.11 mg, 0.2 mmol) in ethanol (5 ml) was added dropwise to a solution of Zn(ClO_4_)_2_.6H_2_O (46.58 mg, 0.1 mmol) in ethanol (2 ml). The mixture was stirred at room temperature for 5 min and then filtered. Colorless crystals of (I) suitable for X-ray analysis were obtained by slow evaporation of the filtrate.

### Refinement

H atoms of water molecule were located in difference Fourier maps and included in the subsequent refinement O–H distance restraints in the range 0.83 (2)–0.85 (4) Å

with *U*_iso_(H)= 1.5*U*_eq_(O). Aromatic H atoms were placed in calculated positions with C—H = 0.93 Å, and refined in riding mode with *U*_iso_(H) = 1.2*U*_eq_(C).

### Crystal data

**Table:**

[Zn(C_15_H_12_N_4_)_2_(H_2_O)_2_](ClO_4_)_2_	*F*(000) = 1632
*M**_r_* = 796.89	*D*_x_ = 1.583 Mg m^−^^3^
Monoclinic, *C*2/*c*	Mo *K*α radiation, λ = 0.71073 Å
Hall symbol: -C 2yc	Cell parameters from 7889 reflections
*a* = 18.687 (3) Å	θ = 2.2–27.9°
*b* = 19.305 (4) Å	µ = 0.96 mm^−^^1^
*c* = 10.8910 (19) Å	*T* = 200 K
β = 121.689 (3)°	Block, colorless
*V* = 3343.2 (11) Å^3^	0.35 × 0.22 × 0.20 mm
*Z* = 4	

### Data collection

**Table:**

Bruker SMART CCD area-detector diffractometer	2940 independent reflections
Radiation source: fine-focus sealed tube	1861 reflections with *I* > 2σ(*I*)
graphite	*R*_int_ = 0.068
Detector resolution: 8.366 pixels mm^-1^	θ_max_ = 25.0°, θ_min_ = 2.1°
phi and ω scans	*h* = −22→21
Absorption correction: multi-scan (*SADABS*; Sheldrick, 1996)	*k* = −18→22
*T*_min_ = 0.775, *T*_max_ = 0.825	*l* = −12→12
7752 measured reflections	

### Refinement

**Table:**

Refinement on *F*^2^	Primary atom site location: structure-invariant direct methods
Least-squares matrix: full	Secondary atom site location: difference Fourier map
*R*[*F*^2^ > 2σ(*F*^2^)] = 0.057	Hydrogen site location: inferred from neighbouring sites
*wR*(*F*^2^) = 0.143	H atoms treated by a mixture of independent and constrained refinement
*S* = 0.92	*w* = 1/[σ^2^(*F*_o_^2^) + (0.0705*P*)^2^] where *P* = (*F*_o_^2^ + 2*F*_c_^2^)/3
2940 reflections	(Δ/σ)_max_ < 0.001
239 parameters	Δρ_max_ = 0.86 e Å^−^^3^
2 restraints	Δρ_min_ = −0.41 e Å^−^^3^

### Special details

**Table:**

Geometry. All e.s.d.'s (except the e.s.d. in the dihedral angle between two l.s. planes) are estimated using the full covariance matrix. The cell e.s.d.'s are taken into account individually in the estimation of e.s.d.'s in distances, angles and torsion angles; correlations between e.s.d.'s in cell parameters are only used when they are defined by crystal symmetry. An approximate (isotropic) treatment of cell e.s.d.'s is used for estimating e.s.d.'s involving l.s. planes.
Refinement. Refinement of *F*^2^ against ALL reflections. The weighted *R*-factor *wR* and goodness of fit *S* are based on *F*^2^, conventional *R*-factors *R* are based on *F*, with *F* set to zero for negative *F*^2^. The threshold expression of *F*^2^ > σ(*F*^2^) is used only for calculating *R*-factors(gt) *etc*. and is not relevant to the choice of reflections for refinement. *R*-factors based on *F*^2^ are statistically about twice as large as those based on *F*, and *R*- factors based on ALL data will be even larger.

### Fractional atomic coordinates and isotropic or equivalent isotropic displacement parameters (Å^2^)

**Table:**

	*x*	*y*	*z*	*U*_iso_*/*U*_eq_	
N1	0.5518 (2)	0.28833 (19)	0.9789 (4)	0.0386 (9)	
C1	0.5611 (3)	0.2247 (2)	1.0527 (5)	0.0419 (12)	
C2	0.5981 (3)	0.2230 (3)	1.1989 (5)	0.0560 (15)	
H2	0.6182	0.2634	1.2530	0.067*	
C3	0.6054 (4)	0.1604 (3)	1.2652 (6)	0.0726 (19)	
H3	0.6305	0.1579	1.3648	0.087*	
C4	0.5750 (4)	0.1018 (3)	1.1816 (7)	0.0750 (19)	
H4	0.5783	0.0591	1.2237	0.090*	
C5	0.5400 (4)	0.1072 (3)	1.0366 (6)	0.0612 (16)	
H5	0.5209	0.0671	0.9811	0.073*	
N2	0.5317 (2)	0.16820 (19)	0.9694 (4)	0.0428 (10)	
C6	0.6036 (3)	0.2977 (2)	0.9218 (5)	0.0382 (11)	
C7	0.6595 (3)	0.3515 (3)	0.9666 (5)	0.0533 (14)	
H7	0.6629	0.3836	1.0331	0.064*	
C8	0.7103 (3)	0.3570 (3)	0.9116 (6)	0.0670 (17)	
H8	0.7474	0.3940	0.9378	0.080*	
C9	0.7060 (3)	0.3072 (3)	0.8166 (6)	0.0598 (16)	
H9	0.7410	0.3096	0.7799	0.072*	
C10	0.6500 (3)	0.2550 (3)	0.7783 (5)	0.0455 (12)	
H10	0.6476	0.2211	0.7155	0.055*	
N3	0.5972 (2)	0.25002 (18)	0.8277 (4)	0.0350 (9)	
Zn1	0.5000	0.17445 (4)	0.7500	0.0376 (3)	
O1W	0.4117 (3)	0.0899 (2)	0.7028 (5)	0.0598 (10)	
H1W	0.387 (4)	0.066 (3)	0.626 (5)	0.10 (3)*	
H2W	0.384 (4)	0.088 (3)	0.742 (6)	0.10 (3)*	
C11	0.5002 (3)	0.3407 (2)	0.9806 (5)	0.0416 (12)	
N4	0.4849 (3)	0.3960 (2)	0.8930 (4)	0.0573 (12)	
C12	0.4310 (3)	0.4453 (3)	0.8870 (6)	0.0569 (15)	
H12	0.4197	0.4841	0.8288	0.068*	
C13	0.3930 (4)	0.4387 (3)	0.9659 (7)	0.0664 (17)	
H13	0.3549	0.4717	0.9595	0.080*	
C14	0.4128 (4)	0.3824 (3)	1.0536 (6)	0.0696 (17)	
H14	0.3890	0.3776	1.1097	0.084*	
C15	0.4662 (3)	0.3336 (2)	1.0602 (5)	0.0494 (13)	
H15	0.4790	0.2953	1.1200	0.059*	
Cl1	0.79396 (8)	0.44302 (7)	0.37729 (14)	0.0488 (4)	
O2	0.8198 (5)	0.3778 (3)	0.3894 (6)	0.200 (4)	
O3	0.7209 (4)	0.4465 (5)	0.3674 (8)	0.222 (4)	
O4	0.8513 (5)	0.4778 (3)	0.4996 (5)	0.159 (3)	
O5	0.7854 (3)	0.4721 (3)	0.2524 (4)	0.1048 (17)	

### Atomic displacement parameters (Å^2^)

**Table:**

	*U*^11^	*U*^22^	*U*^33^	*U*^12^	*U*^13^	*U*^23^
N1	0.048 (2)	0.032 (2)	0.036 (2)	0.0059 (18)	0.022 (2)	−0.0005 (17)
C1	0.051 (3)	0.040 (3)	0.041 (3)	0.015 (2)	0.028 (3)	0.008 (2)
C2	0.071 (4)	0.056 (4)	0.044 (3)	0.023 (3)	0.032 (3)	0.008 (3)
C3	0.103 (5)	0.078 (5)	0.049 (4)	0.027 (4)	0.049 (4)	0.018 (3)
C4	0.119 (6)	0.057 (4)	0.074 (4)	0.027 (4)	0.067 (4)	0.029 (3)
C5	0.094 (5)	0.040 (3)	0.071 (4)	0.012 (3)	0.058 (4)	0.014 (3)
N2	0.060 (3)	0.034 (2)	0.048 (3)	0.007 (2)	0.037 (2)	0.0080 (19)
C6	0.034 (3)	0.035 (3)	0.033 (3)	0.003 (2)	0.009 (2)	0.007 (2)
C7	0.050 (3)	0.047 (3)	0.043 (3)	−0.003 (3)	0.011 (3)	0.002 (3)
C8	0.041 (3)	0.065 (4)	0.067 (4)	−0.016 (3)	0.009 (3)	0.015 (3)
C9	0.034 (3)	0.076 (4)	0.064 (4)	0.002 (3)	0.022 (3)	0.024 (3)
C10	0.045 (3)	0.048 (3)	0.046 (3)	0.007 (3)	0.027 (3)	0.011 (2)
N3	0.035 (2)	0.033 (2)	0.037 (2)	0.0035 (17)	0.0185 (19)	0.0030 (17)
Zn1	0.0483 (5)	0.0296 (4)	0.0440 (5)	0.000	0.0307 (4)	0.000
O1W	0.077 (3)	0.049 (3)	0.071 (3)	−0.019 (2)	0.051 (3)	−0.009 (2)
C11	0.042 (3)	0.034 (3)	0.033 (3)	0.009 (2)	0.009 (2)	−0.002 (2)
N4	0.054 (3)	0.056 (3)	0.048 (3)	0.008 (2)	0.018 (2)	−0.004 (2)
C12	0.055 (4)	0.049 (3)	0.055 (4)	0.015 (3)	0.020 (3)	0.005 (3)
C13	0.071 (4)	0.056 (4)	0.078 (4)	0.023 (3)	0.044 (4)	0.000 (3)
C14	0.087 (5)	0.069 (4)	0.069 (4)	0.017 (4)	0.053 (4)	0.006 (3)
C15	0.074 (4)	0.036 (3)	0.044 (3)	0.021 (3)	0.035 (3)	0.010 (2)
Cl1	0.0522 (8)	0.0533 (9)	0.0487 (8)	0.0061 (6)	0.0319 (7)	0.0047 (6)
O2	0.349 (10)	0.089 (4)	0.096 (4)	0.114 (6)	0.070 (5)	0.005 (3)
O3	0.115 (5)	0.399 (12)	0.223 (8)	0.073 (6)	0.138 (6)	0.137 (8)
O4	0.281 (8)	0.097 (4)	0.063 (3)	−0.065 (5)	0.066 (4)	−0.025 (3)
O5	0.093 (3)	0.166 (5)	0.057 (3)	−0.016 (3)	0.040 (3)	0.027 (3)

### Geometric parameters (Å, °)

**Table:**

N1—C11	1.404 (5)	C10—H10	0.9300
N1—C6	1.411 (6)	N3—Zn1	2.128 (4)
N1—C1	1.428 (5)	Zn1—N3^i^	2.128 (4)
C1—N2	1.338 (6)	Zn1—N2^i^	2.142 (4)
C1—C2	1.364 (6)	Zn1—O1W	2.182 (4)
C2—C3	1.376 (7)	Zn1—O1W^i^	2.182 (4)
C2—H2	0.9300	O1W—H1W	0.85 (4)
C3—C4	1.374 (8)	O1W—H2W	0.83 (2)
C3—H3	0.9300	C11—C15	1.325 (6)
C4—C5	1.359 (7)	C11—N4	1.358 (6)
C4—H4	0.9300	N4—C12	1.364 (6)
C5—N2	1.352 (6)	C12—C13	1.379 (7)
C5—H5	0.9300	C12—H12	0.9300
N2—Zn1	2.142 (4)	C13—C14	1.364 (8)
C6—N3	1.335 (5)	C13—H13	0.9300
C6—C7	1.368 (7)	C14—C15	1.346 (7)
C7—C8	1.366 (7)	C14—H14	0.9300
C7—H7	0.9300	C15—H15	0.9300
C8—C9	1.384 (8)	Cl1—O3	1.314 (5)
C8—H8	0.9300	Cl1—O2	1.330 (5)
C9—C10	1.352 (7)	Cl1—O4	1.369 (5)
C9—H9	0.9300	Cl1—O5	1.401 (4)
C10—N3	1.352 (5)		
			
C11—N1—C6	123.2 (4)	N3—Zn1—N2^i^	99.25 (14)
C11—N1—C1	119.6 (4)	N3^i^—Zn1—N2^i^	85.21 (14)
C6—N1—C1	116.7 (4)	N3—Zn1—N2	85.21 (14)
N2—C1—C2	123.2 (5)	N3^i^—Zn1—N2	99.25 (14)
N2—C1—N1	115.8 (4)	N2^i^—Zn1—N2	173.5 (2)
C2—C1—N1	121.0 (5)	N3—Zn1—O1W	171.28 (15)
C1—C2—C3	118.9 (5)	N3^i^—Zn1—O1W	92.16 (15)
C1—C2—H2	120.6	N2^i^—Zn1—O1W	87.87 (16)
C3—C2—H2	120.6	N2—Zn1—O1W	87.30 (16)
C4—C3—C2	118.9 (5)	N3—Zn1—O1W^i^	92.16 (15)
C4—C3—H3	120.6	N3^i^—Zn1—O1W^i^	171.28 (15)
C2—C3—H3	120.6	N2^i^—Zn1—O1W^i^	87.30 (16)
C5—C4—C3	119.0 (5)	N2—Zn1—O1W^i^	87.87 (16)
C5—C4—H4	120.5	O1W—Zn1—O1W^i^	83.1 (2)
C3—C4—H4	120.5	Zn1—O1W—H1W	126 (4)
N2—C5—C4	123.0 (5)	Zn1—O1W—H2W	120 (5)
N2—C5—H5	118.5	H1W—O1W—H2W	109 (6)
C4—C5—H5	118.5	C15—C11—N4	123.1 (4)
C1—N2—C5	116.9 (4)	C15—C11—N1	120.2 (4)
C1—N2—Zn1	119.0 (3)	N4—C11—N1	116.7 (4)
C5—N2—Zn1	122.6 (3)	C11—N4—C12	117.4 (4)
N3—C6—C7	122.7 (5)	N4—C12—C13	121.1 (5)
N3—C6—N1	116.3 (4)	N4—C12—H12	119.5
C7—C6—N1	120.9 (5)	C13—C12—H12	119.5
C8—C7—C6	118.6 (5)	C14—C13—C12	118.0 (5)
C8—C7—H7	120.7	C14—C13—H13	121.0
C6—C7—H7	120.7	C12—C13—H13	121.0
C7—C8—C9	119.5 (5)	C15—C14—C13	121.2 (5)
C7—C8—H8	120.2	C15—C14—H14	119.4
C9—C8—H8	120.2	C13—C14—H14	119.4
C10—C9—C8	118.7 (5)	C11—C15—C14	119.3 (5)
C10—C9—H9	120.6	C11—C15—H15	120.4
C8—C9—H9	120.6	C14—C15—H15	120.4
N3—C10—C9	122.5 (5)	O3—Cl1—O2	111.4 (6)
N3—C10—H10	118.7	O3—Cl1—O4	107.6 (5)
C9—C10—H10	118.7	O2—Cl1—O4	108.1 (4)
C6—N3—C10	117.8 (4)	O3—Cl1—O5	108.4 (4)
C6—N3—Zn1	119.5 (3)	O2—Cl1—O5	109.0 (4)
C10—N3—Zn1	122.6 (3)	O4—Cl1—O5	112.4 (3)
N3—Zn1—N3^i^	93.45 (19)		
			
C11—N1—C1—N2	115.9 (5)	C9—C10—N3—Zn1	173.1 (4)
C6—N1—C1—N2	−71.3 (5)	C6—N3—Zn1—N3^i^	56.2 (3)
C11—N1—C1—C2	−64.4 (6)	C10—N3—Zn1—N3^i^	−119.1 (4)
C6—N1—C1—C2	108.4 (5)	C6—N3—Zn1—N2^i^	141.9 (3)
N2—C1—C2—C3	−0.5 (8)	C10—N3—Zn1—N2^i^	−33.4 (4)
N1—C1—C2—C3	179.8 (5)	C6—N3—Zn1—N2	−42.8 (3)
C1—C2—C3—C4	−0.1 (9)	C10—N3—Zn1—N2	141.9 (4)
C2—C3—C4—C5	1.1 (9)	C6—N3—Zn1—O1W^i^	−130.5 (3)
C3—C4—C5—N2	−1.6 (9)	C10—N3—Zn1—O1W^i^	54.2 (4)
C2—C1—N2—C5	0.1 (7)	C1—N2—Zn1—N3	30.8 (3)
N1—C1—N2—C5	179.8 (4)	C5—N2—Zn1—N3	−135.2 (4)
C2—C1—N2—Zn1	−166.8 (4)	C1—N2—Zn1—N3^i^	−61.9 (3)
N1—C1—N2—Zn1	12.9 (5)	C5—N2—Zn1—N3^i^	132.0 (4)
C4—C5—N2—C1	1.0 (8)	C1—N2—Zn1—O1W	−153.6 (4)
C4—C5—N2—Zn1	167.3 (4)	C5—N2—Zn1—O1W	40.3 (4)
C11—N1—C6—N3	−129.6 (4)	C1—N2—Zn1—O1W^i^	123.2 (4)
C1—N1—C6—N3	57.8 (5)	C5—N2—Zn1—O1W^i^	−42.9 (4)
C11—N1—C6—C7	53.0 (6)	C6—N1—C11—C15	−166.6 (5)
C1—N1—C6—C7	−119.5 (5)	C1—N1—C11—C15	5.8 (7)
N3—C6—C7—C8	0.7 (7)	C6—N1—C11—N4	16.3 (6)
N1—C6—C7—C8	177.9 (4)	C1—N1—C11—N4	−171.4 (4)
C6—C7—C8—C9	−2.2 (8)	C15—C11—N4—C12	−0.8 (7)
C7—C8—C9—C10	1.5 (8)	N1—C11—N4—C12	176.2 (4)
C8—C9—C10—N3	0.9 (8)	C11—N4—C12—C13	−0.7 (8)
C7—C6—N3—C10	1.6 (7)	N4—C12—C13—C14	1.9 (9)
N1—C6—N3—C10	−175.7 (4)	C12—C13—C14—C15	−1.7 (9)
C7—C6—N3—Zn1	−174.0 (3)	N4—C11—C15—C14	1.0 (8)
N1—C6—N3—Zn1	8.7 (5)	N1—C11—C15—C14	−175.9 (5)
C9—C10—N3—C6	−2.4 (7)	C13—C14—C15—C11	0.3 (9)

Symmetry codes: (i) −*x*+1, *y*, −*z*+3/2.

### Hydrogen-bond geometry (Å, °)

**Table:**

*D*—H···*A*	*D*—H	H···*A*	*D*···*A*	*D*—H···*A*
C3—H3···O2^ii^	0.93	2.43	3.336 (8)	166
O1W—H2W···O5^iii^	0.83 (2)	2.23 (4)	2.939 (6)	143 (6)
O1W—H1W···O4^iv^	0.85 (4)	2.07 (5)	2.868 (6)	158 (6)

Symmetry codes: (ii) −*x*+3/2, −*y*+1/2, −*z*+2; (iii) *x*−1/2, −*y*+1/2, *z*+1/2; (iv) *x*−1/2, *y*−1/2, *z*.

## References

[bb1] Brandenburg, K. (1999). *DIAMOND* Crystal Impact GbR, Bonn, Germany.

[bb2] Bruker (2007). *SMART* and *SAINT* Bruker AXS Inc., Madison, Wisconsin, USA.

[bb3] Liu, W., Hassan, A. & Wang, S. (1997). *Organometallics*, **16**, 4257–4259.

[bb4] Sheldrick, G. M. (1996). *SADABS* University of Göttingen, Germany.

[bb5] Sheldrick, G. M. (2008). *Acta Cryst.* A**64**, 112–122. 10.1107/S0108767307043930 18156677

[bb6] Yang, W., Schmider, H., Wu, Q., Zhang, Y. & Wang, S. (1999). *Inorg. Chem.* **39**, 2397–2404. 10.1021/ic991436m 12526502

